# Biosynthesis and possible functions of inositol pyrophosphates in plants

**DOI:** 10.3389/fpls.2015.00067

**Published:** 2015-02-12

**Authors:** Sarah P. Williams, Glenda E. Gillaspy, Imara Y. Perera

**Affiliations:** ^1^Biochemistry, Virginia Polytechnic and State UniversityBlacksburg, VA, USA; ^2^Plant and Microbial Biology, North Carolina State UniversityRaleigh, NC, USA

**Keywords:** plant inositol signaling, inositol hexakisphosphate, VIP, inositol pyrophosphate, energy metabolism

## Abstract

Inositol phosphates (InsPs) are intricately tied to lipid signaling, as at least one portion of the inositol phosphate signaling pool is derived from hydrolysis of the lipid precursor, phosphatidyl inositol (4,5) bisphosphate. The focus of this review is on the inositol pyrophosphates, which are a novel group of InsP signaling molecules containing diphosphate or triphosphate chains (i.e., PPx) attached to the inositol ring. These PPx-InsPs are emerging as critical players in the integration of cellular metabolism and stress signaling in non-plant eukaryotes. Most eukaryotes synthesize the precursor molecule, myo-inositol (1,2,3,4,5,6)-hexakisphosphate (InsP6), which can serve as a signaling molecule or as storage compound of inositol, phosphorus, and minerals (referred to as phytic acid). Even though plants produce huge amounts of precursor InsP6 in seeds, almost no attention has been paid to whether PPx-InsPs exist in plants, and if so, what roles these molecules play. Recent work has delineated that Arabidopsis has two genes capable of PP-InsP5 synthesis, and PPx-InsPs have been detected across the plant kingdom. This review will detail the known roles of PPx-InsPs in yeast and animal systems, and provide a description of recent data on the synthesis and accumulation of these novel molecules in plants, and potential roles in signaling.

*Myo-*inositol (inositol) signaling is much like a language in that each molecular species used in the pathway, whether lipid or soluble in nature, can convey specific information to the cell, like a word. In this analogy, each combination of different numbers and positions of phosphates on the inositol ring and the presence of diacylglycerol linked via the C1 of inositol, also convey unique information (see Figure 1). Comprehensive analyses of both inositol and inositol phospholipids in signaling have been previously reviewed (Van Leeuwen et al., 2004; Gillaspy, 2011; Heilmann and Heilmann, 2014), so this review focuses on new inositol signaling molecules, the di-phospho (PP) and tri-phospho (PPP) inositol phosphates (PPx-InsPs), also known as inositol pyrophosphates. These high-energy molecules have been studied in non-plant eukaryotes, however, their existence and role in plants is newly emerging. The main questions regarding PPx-InsPs are: can these molecules be detected in plants, how are they synthesized and what type of information do they convey? Recent work addressing these questions will be discussed in the broader context of understanding how PPx-InsPs function in eukaryotes.

**Figure 1:**
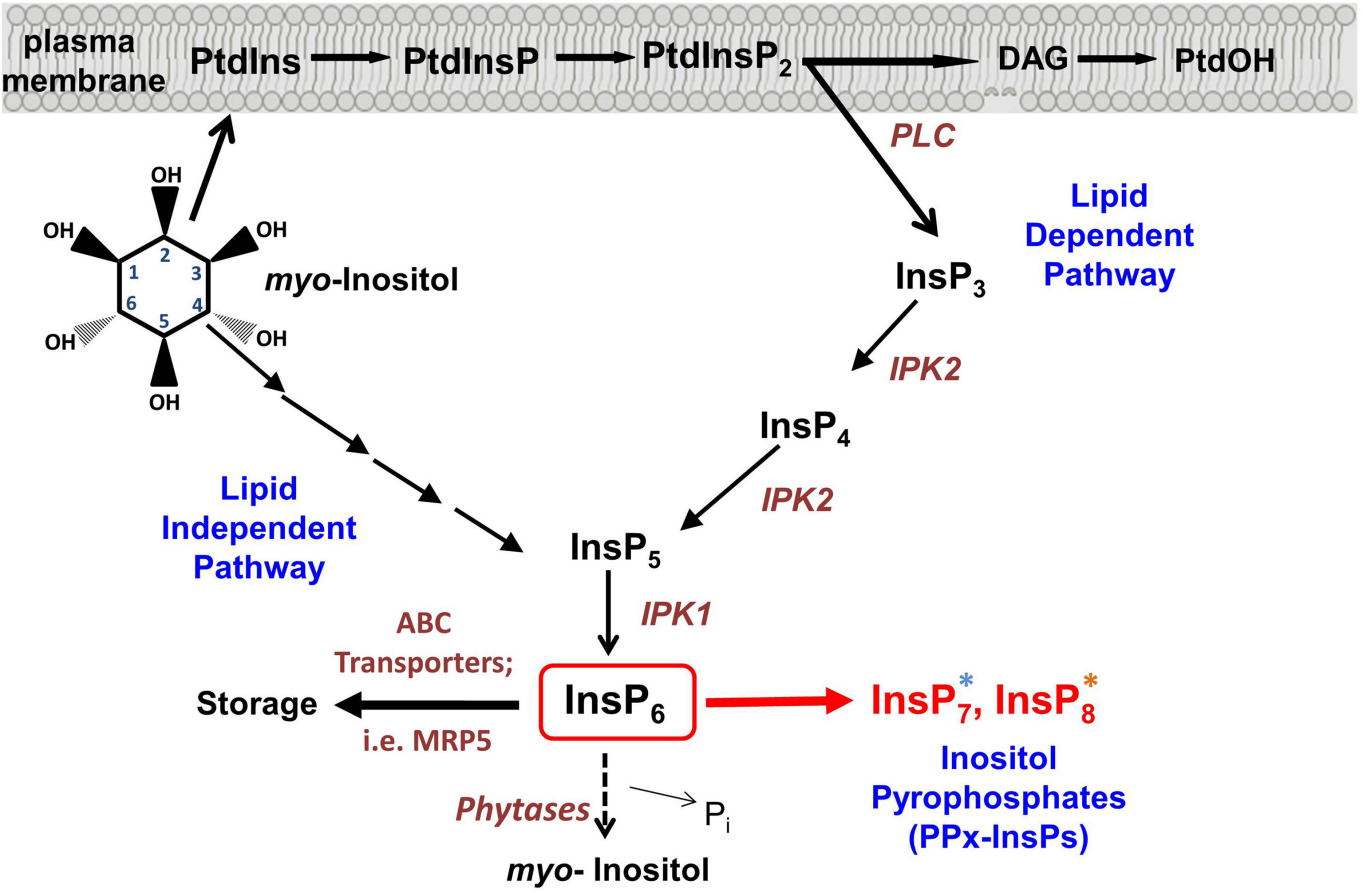
**Synthesis of inositol pyrophosphate**. Overview of the Inositol phosphate pathway, including both lipid dependent and lipid independent routes for synthesis of InsP_6_. Inositol Pyrophosphate (PPx-InsP) synthesis is indicated in red. Major lipid and inositol species are indicated in black and key enzymes are indicated in brown. A more detailed outline of PPx-InsP synthesis is depicted in **Figure 2**. The blue and orange asterisks correspond to the colored boxes in **Figure 2**.

## History and structure of PPx-InsPs

**Figure 2:**
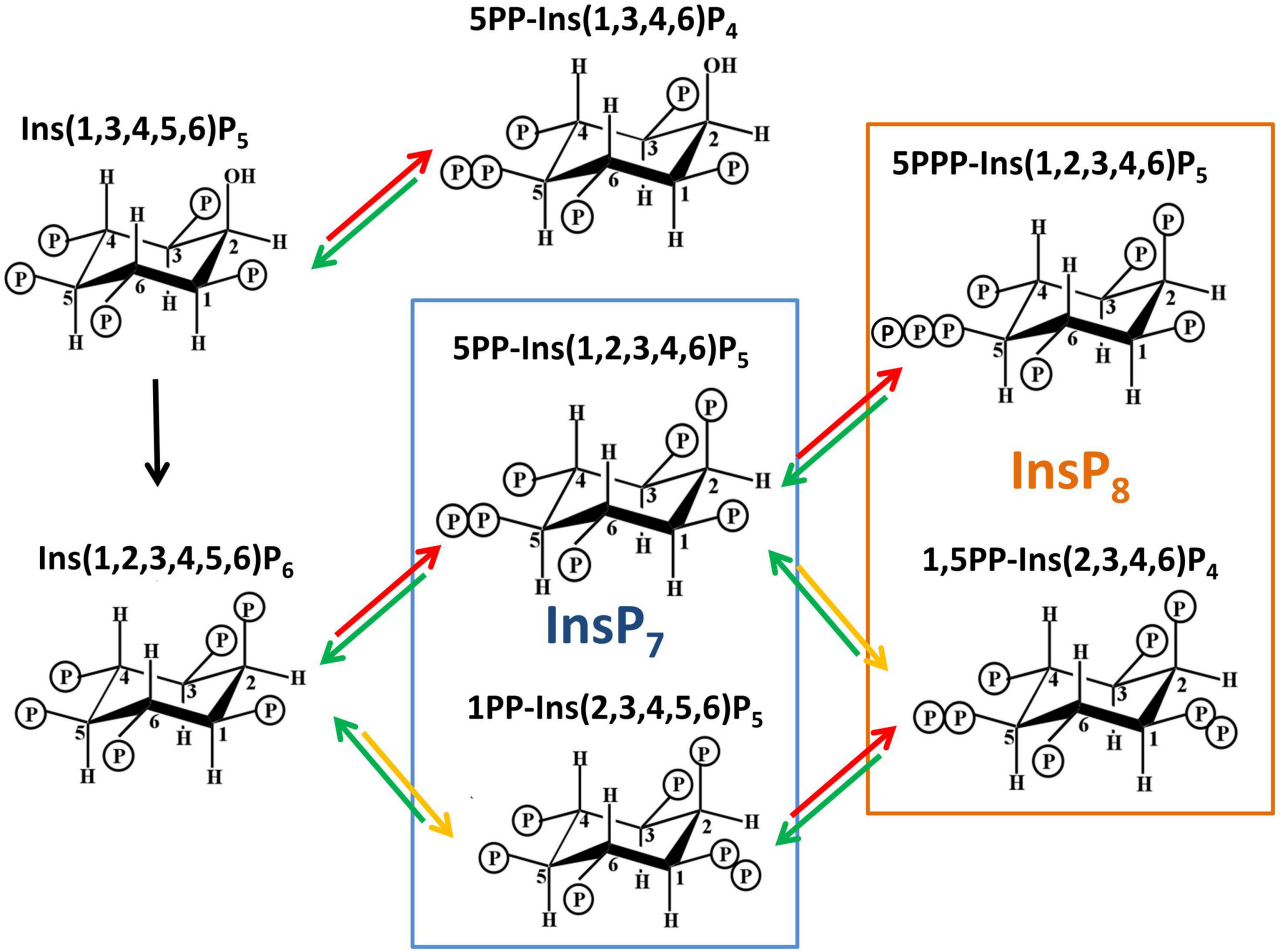
**Structures of PPx-InsPs and pathway of proposed synthesis**. The unboxed area is the last step in InsP_6_ synthesis, catalyzed by the IPK1 enzyme in plants. The boxed areas in blue and orange indicate InsP_7_ and InsP_8_ synthesis. The colored arrows indicate known enzymes in yeast. Red arrows indicate KCS1 activity, while yellow arrows indicate VIP activity. The green arrows indicate phosphatase activity by DDP1.

PPx-InsPs were first identified in Dictyostelium in 1993 (Glennon and Shears, 1993; Hawkins et al., 1993; Menniti et al., 1993; Stephens et al., 1993). They are similar to ATP and polyphosphates in that they contain a linear chain of two (PP) or three (PPP) phosphates separated by pyrophosphate bonds, linked to an InsP molecule (see Figure 2).

The PPx moieties at one or more positions on the inositol ring in PPx-InsPs are synthesized from InsP_5_ or InsP_6_ (Menniti et al., 1993; Shears et al., 2011), resulting in InsPs containing seven or eight phosphates (i.e., InsP_7_ and InsP_8_). The high energy pyrophosphate bonds present in PPx-InsPs may serve as a way to store energy in the cell, with the standard free energy of hydrolysis of InsP_7_ and InsP_8_ higher than that of ADP and ATP, respectively (Stephens et al., 1993). Indeed, the initial role proposed for PPx-InsPs was simply as a high energy molecules, as they can be broken down to generate ATP (Voglmaier et al., 1996; Huang et al., 1998). However, PPx-InsPs are present at very low amounts and they have high turnover rates, suggesting that they serve as more than energy storage molecules (Glennon and Shears, 1993; Menniti et al., 1993). Recently new physiological roles have been discovered for PPx-InsPs, supporting their role as dynamic and important signaling molecules.

Only a few of the theoretically possible PPx-InsP structures have been confirmed. The naming convention is to describe the position and number of the pyrophosphates first, followed by the name of the InsP. For example, Dictyostelium contains 6PP-InsP_5_ and 5PP-InsP_5_, and these contain a pyrophosphate on the 6th and 5th carbons of InsP_5_, respectively. Dictyostelium also contains 5,4/6(PP)_2_-InsP_4_ or 1/3,5(PP)_2_-InsP_4_, and in these cases the slash indicates one of the pyrophosphates present can occur at *either* of two carbons (i.e., at the C4 or C6 position, or at C1 or C3, respectively). The ratios of these different PPx-InsPs differ in various Dictyostelium species examined (Laussmann et al., 1997, 1998). Another member of the Amoebazoa kingdom, *Entamoeba histolytica*, has further diversity in that a PPx-InsP was identified containing *neo*-inositol, rather than *myo-*inositol (Martin et al., 2000). This difference could produce even more diversity in the language of InsPs, but it is not yet known if this occurs in other organisms.

In Dictyostelium and the animal kingdom, PPx-InsPs synthesized from InsP_5_ exist (Laussmann et al., 1997, 1998; Draskovic et al., 2008), however at present there is no data indicating they are found in plants. This review will focus on the PPx-InsP species synthesized from InsP_6_. The most abundant InsP_7_ isomer has been confirmed through NMR as 5PP-InsP_5_ (Mulugu et al., 2007; Draskovic et al., 2008). A second InsP_7_ was first speculated to be pyrophosphorylated at C4 or C6 (i.e., 4/6), but was later conclusively identified as 1/3PP-InsP_5_ (Lin et al., 2009). Recent work in animals has shown that the 1- rather than 3- position is phosphorylated, thus 1PP-InsP_5_ is likely to be the second type of InsP_7_ present in animals (Wang et al., 2012). Given this, we use 1PP-InsP_5_ as the updated nomenclature for this second molecular species of InsP_7_. Studies in yeast and humans identified that both of these InsP_7_ molecules are present. The InsP_8_ species confirmed are 1,5(PP)_2_-InsP_4_ (*in vivo*) and 5PPP-InsP_5_(*in vitro*) (Draskovic et al., 2008; Lin et al., 2009).

## Methods used to detect PPx-InsPs

Several methods have been used to detect PPx-InsPs, each having distinct strengths and limitations. The most common method is to introduce a radiolabeled precursor, often ^3^H-*myo*-inositol or ^3^H-InsP_6_, followed by HPLC separation of InsP species produced after a given time (Azevedo and Saiardi, 2006). This method is very sensitive, but labor-intensive, and while it can resolve isomers of the lower InsPs, currently it is not possible to separate different PPx-InsP isomers. As well, one is limited to analysis of cells/tissues that can take up the radiolabelled precursor. However, this method has an advantage in that one can be fairly certain of the identity of the resulting labeled peaks on the HPLC chromatograms. A nonradioactive high-performance anion-exchange chromatographic method based on metal dye detection (MDD)-HPLC can also be used to detect PPx-InsPs. The advantage of this method is that it can separate different isomers of InsP_7_, however this method requires a 3 pump HPLC unit which increases the complexity of the system and limits its use (Mayr, 1988). Another method of separation of InsPs is thin layer chromatography, which utilizes either radiolabeling or dye for detection of InsP species (Otto et al., 2007). This method, in general, does not have great sensitivity, and is most often used with purified PPx-InsPs.

Since acidic conditions can cause the degradation of PPx-InsPs, HPLC analyses may underestimate the amount of PPx-InsPs present (Losito et al., 2009). A new method developed to limit exposure of extracted PPx-InsPs to acid buffers is polyacrylamide gel separation by electrophoresis (PAGE), and subsequent staining with either DAPI or toluidine blue to detect InsPs (Losito et al., 2009). The PAGE technique is sensitive enough to visualize PPx-InsPs from cell/tissue extracts, and it can separate different InsP_7_ and InsP_8_ isomers. However, its distinct advantage is that it may allow for a better estimation of PPx-InsP abundance because of the speed of analysis. The disadvantage of using PAGE is that conclusive identification of stained “bands” as PPx-InsPs is best verified with a separate technology, as other molecules could be present and give rise to bands. It is important to note that co-migration with InsP and PPx-InsP standards is required for all of these methods, and follow-up NMR is needed to conclusively identify the specific structure of PPx-InsP species.

## Plants contain PPx-InsPs

Plants have large amounts of one of the precursors to PPx-InsPs, InsP_6_, which is well studied as a phosphorous storage molecule (Raboy, 2003). Given this, it seems likely that plants also synthesize the PPx-InsPs. Previous studies had noted InsP molecules more polar than InsP_6_ in barley, duckweed, and potato (Brearley and Hanke, 1996; Flores and Smart, 2000; Lemtiri-Chlieh et al., 2000; Dorsch et al., 2003). Acting on this information, we recently utilized both HPLC separation of radiolabelled plant tissues and PAGE to delineate the presence of InsP_7_ and InsP_8_ in higher plants including Arabidopsis, *Camelina sativa*, cotton, and maize (Desai et al., 2014). These two methods provided a complementary analysis of higher phosphorylated InsPs, including the PPx-InsPs. Since PPx-InsPs are low abundance molecules, it is not surprising that Arabidopsis seeds were found to contain less than 2% of the total inositol pool as inositol pyrophosphates (1.33% InsP_7_, 0.24% InsP_8_). Vegetative tissue from Arabidopsis was also analyzed and PPx-InsPs were found in both seedlings (0.64% InsP_7_, 0.14% InsP_8_) and mature leaves (1.00% InsP_7_). InsP_7_ was detected in other plant species as well, including another member of the Brassica family, *Camelina sativa* (1.40% in seedlings), and an unrelated dicot, cotton (*Gossypium hirsutum*) in the leaves and in shoots and roots of seedlings. PAGE analysis was used to detect PPx-InsPs in both Arabidopsis and maize seed in this same work (Desai et al., 2014). These findings indicate that PPx-InsPs may play a role during the plant life cycle throughout the plant kingdom, both in monocots and dicots.

Critical to our detection of the PPx-InsPs in plants was the use of a mutant containing elevated InsP_7_ and InsP_8_. The Multidrug Resistance associated Protein 5 (MRP5) is a high affinity ABC-binding cassette transporter that specifically binds to InsP_6_ (Nagy et al., 2009) (Figure 1). Studies on MRP5 have indicated the likely role of this transporter is in moving InsP_6_ into the storage vacuole (Nagy et al., 2009). The subcellular localization of MRP5 has been reported as both the plasma membrane (Suh et al., 2007) and the vacuolar membrane (Nagy et al., 2009), however it has been suggested that the plasma membrane localization is an artifact resulting from ectopic expression (Nagy et al., 2009). MRP5 was first identified as an important player in stomatal responses, since guard cells of the loss-of-function *mrp5* mutant are insensitive to ABA and Ca^2+^ (Klein et al., 2003). This alteration in guard cell function results in reduction of water loss and use, allowing *mrp5* mutants some resistance to drought (Klein et al., 2003). The maize paralogue of MRP5 (called MRP4), results in decreased levels of InsP_6_ in seeds when mutated (Shi et al., 2007; Nagy et al., 2009). Our recent study showed that in addition to decreased levels of InsP_6_, *mrp5* mutants have elevated levels of InsP_7_ and InsP_8_ in seeds (Desai et al., 2014). These changes are less striking in vegetative tissue, perhaps as a result of overall lower levels of InsP_6_ (Desai et al., 2014), or reduced MRP5 expression (Nagy et al., 2009) in vegetative tissues. The guard cell phenotype of *mrp5* mutants has been attributed to an increase in cytosolic InsP_6_, which could mobilize Ca^2+^, leading to inhibition of inward rectifying K^+^ channels, and changes in turgor pressure resulting in a decreased stomatal aperture (Lemtiri-Chlieh et al., 2000, 2003; Nagy et al., 2009). With the identification of elevated PPx-InsPs in *mrp5* mutants, an alternative hypothesis for alterations in *mrp5* guard cell signaling is that changes in InsP_7_ and InsP_8_ may be involved.

It should be noted that the recent study identifying PPx-InsPs in plants was not able to discern the enantiomers present (Desai et al., 2014). Thus, it is not known whether plants contain 5PP-InsP_7_ or 1PP-InsP_7_ similar to yeast and animals, or (4/6)PP-InsP_7_, similar to Dictyostelium. Efforts were made to purify the plant PPx-InsPs, however no informative NMR data was obtained (Desai et al., 2014). The identity of the plant isomers is key, and in itself may yield insights into the pathway, as different types of enzymes in other organisms give rise to specific PPx-InsP isomers. The following section describes this relationship between PPx-InsP synthesis and isomers in detail.

## Synthesis of PPx-InsPs by KCS1/IP6K enzymes

**Figure 3:**
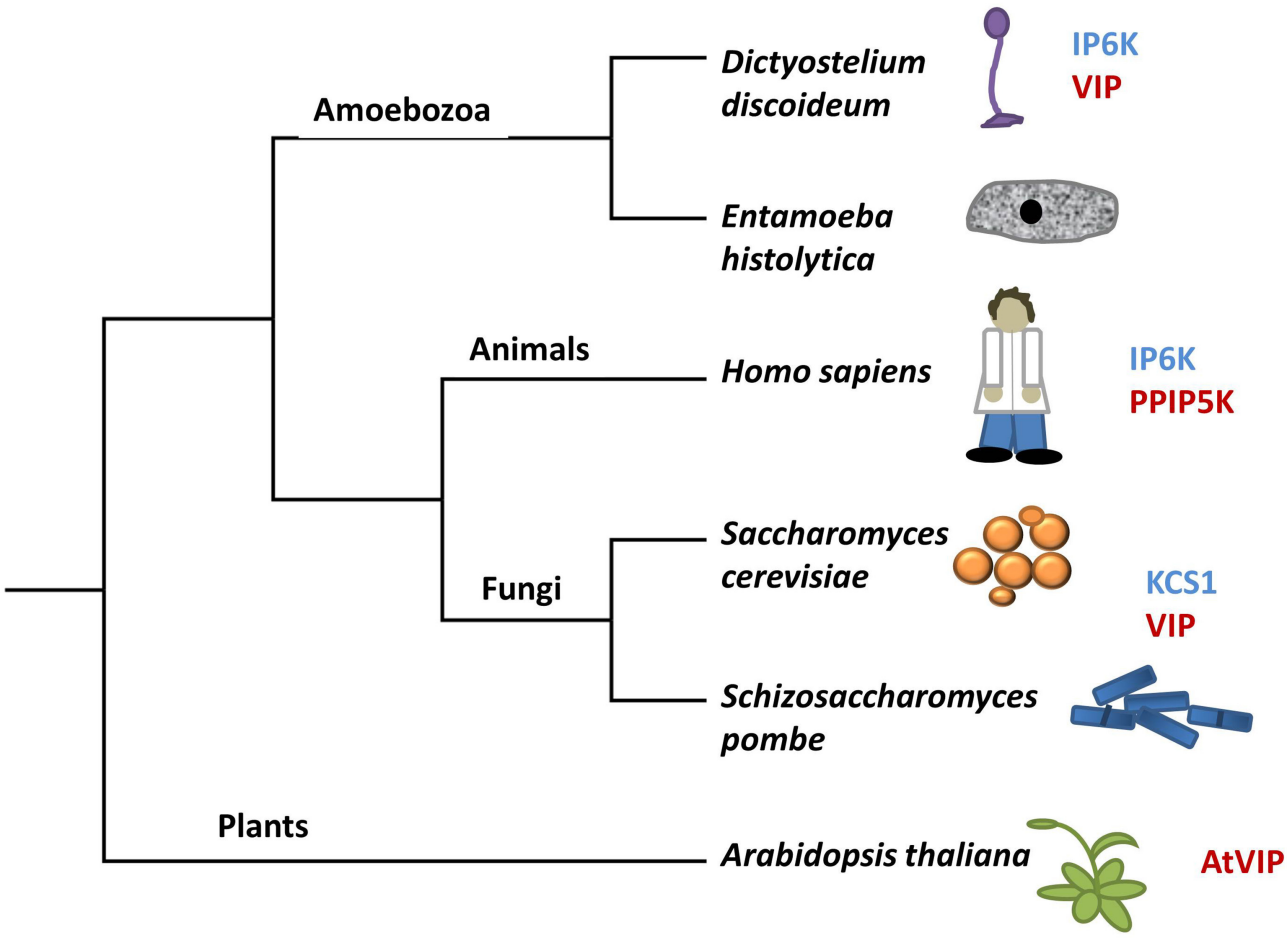
**A modified tree of life indicating the composition of genes in different species that encode kinases capable of phosphorylating InsP_6_**. Genes in blue have sequence identity with KCS1, whereas genes in red have sequence identity with VIP. The tree depicts evolutionary relationships between groups discussed in the review.

There are two classes of genes shown to encode enzymes required for synthesis of PPx-InsPs. Figure 3 shows the presence and names of these genes in species relevant to this review. These two classes of genes encode distinct enzymes that catalyze the addition of pyrophosphates at specific positions on the inositol ring (Figure 2). The first class is named the InsP_6_ kinases (IP6Ks), and the kinase activity of these enzymes phosphorylates the 5-position of InsP_5_, InsP_6_, and InsP_7_, yielding 5PP-InsP_4_ or 5PP-InsP_5_ and two possible forms of InsP_8_: 1/3,5PP-InsP_5_ and 5PPP-InsP_5_ (Draskovic et al., 2008). In yeast, this class of enzymes is named KCS1, and was first identified in a suppressor screen of the yeast Protein Kinase C (*pkc1*) mutant (Huang and Symington, 1995). *Kcs1* encodes a protein closely related to the bZIP family of transcription factors, although analysis of its two potential leucine zipper motifs indicates the secondary alpha-helical structure for DNA binding is not formed (Huang and Symington, 1995). Instead, the altered structure of this alpha helix in addition to a two-turn 3_10_ helix, forms a pocket for InsP_6_ binding (Wang et al., 2014).

Under low energy conditions, KCS1 can generate ATP from InsP_6_ (Wundenberg et al., 2014). This dual function of KCS1 to both degrade InsP_6_ and generate InsP_7_ presents the possibility of KCS1 acting as an ATP/ADP ratio sensor (Wundenberg et al., 2014). Given the considerable amount of InsP_6_ in plants, if present, a KCS1-like enzyme could generate a significant source of ATP under low energy conditions. However, sequence homology searches using the yeast KCS1 and human IP6K proteins indicate that there are no KCS1/IP6K homologs in plants (Bennett et al., 2006; Desai et al., 2014).

In the absence of a plant KCS1/IP6K, one might expect that 5PP-InsP_5_ could not be synthesized. However, there is the possibility of another InsP kinase in plants acting as a KCS1/IP6K in the synthesis of 5PP-InsP_5_. The larger InsP kinase family (Pfam PF03770) has a PxxxDxKxG (“PDKG”) catalytic motif and includes the InsP_3_ kinases (IP3Ks), IP6K, and inositol polyphosphate multikinases (IPMKs). Recent phylogenetic studies on these enzymes has suggested that the InsP kinase family common ancestor is an IP6K precursor (Bennett et al., 2006). The rationale is that the substrate-binding pocket for InsP_6_ is larger, and from this common ancestor, the binding pocket would shrink to become more specific for other InsPs (Wang et al., 2014). Not all extant species have developed kinases solely acting on InsP_3_: *Entamoeba histolytica* still has an IP3K which retains IP6K activity (Wang et al., 2014), presenting the possibility that if plants have a KCS1/IP6K, it may be distinct from that of yeast and mammals.

## Synthesis of PPx-InsPs by VIP/PPIP5K enzymes

The second class of enzymes capable of synthesizing PPx-InsPs are the VIPs, which are also known as diphosphoinositol pentakisphosphate kinases (PPIP5Ks) in animals (Shears et al., 2013) (Figures 2, 3). In quick succession, two groups identified VIPs in yeast and mammalian cells (Choi et al., 2007; Fridy et al., 2007; Mulugu et al., 2007). The name PPIP5K originated as scientists were looking for an enzyme capable of phosphorylating PP-InsP_5_ to produce the InsP_8_, which had been observed in mammalian cell extracts (Stephens et al., 1993). The PPIP5K that was identified has a higher affinity for InsP_7_ than InsP_6_ (Choi et al., 2007). These enzymes produce a structurally distinct InsP_7_ with recent NMR work delineating 1PP-InsP_7_ as the product (Wang et al., 2012). These enzymes can also phosphorylate 5PP-InsP_5_ to produce 1,5(PP)_2_-InsP_4_ (Figure 2) or speculatively, even 1PPP-InsP_5_.

**Figure 4:**
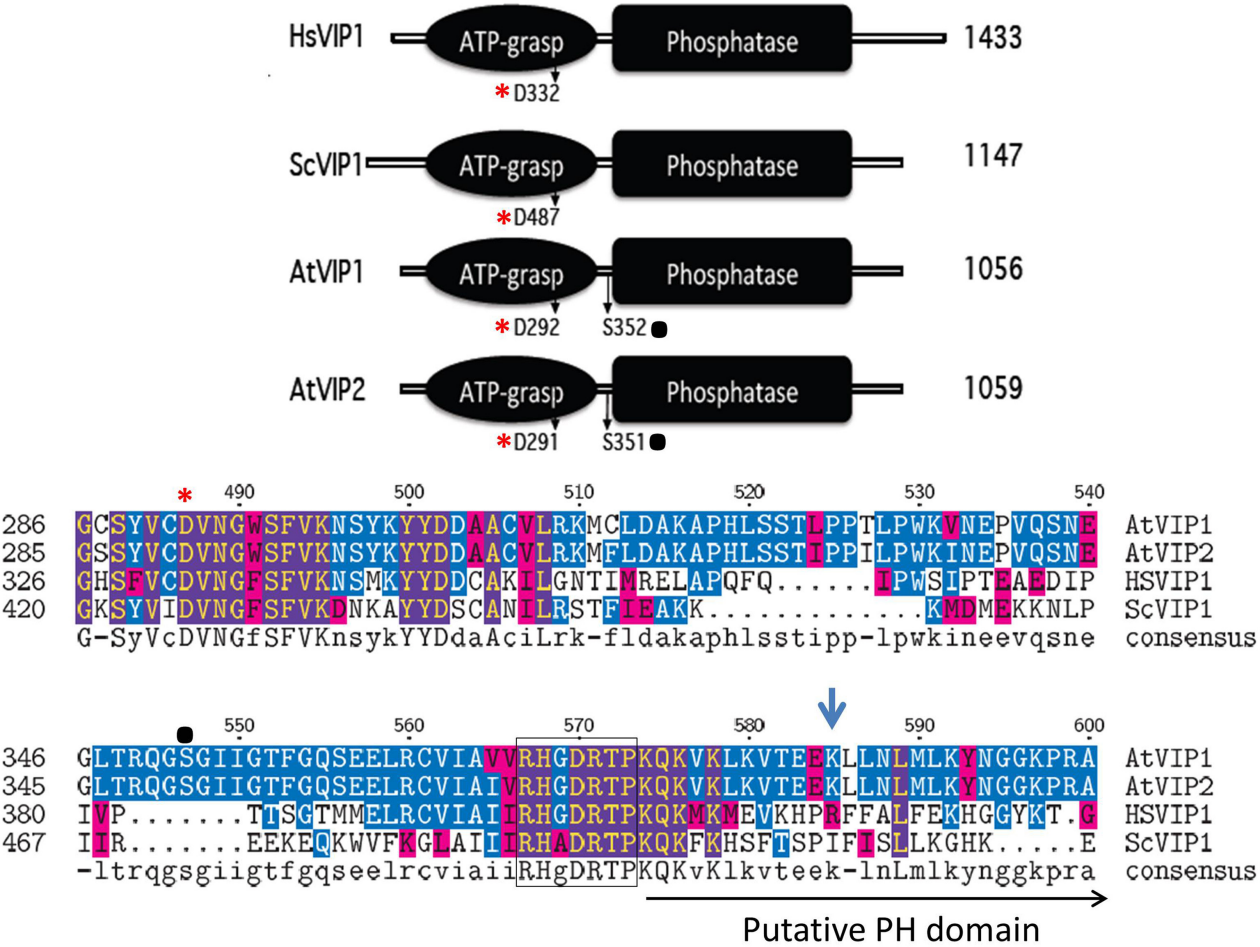
**Schematic alignment of the kinase and phosphatase domains of VIPs**. The ATP grasp/RimK/ kinase (ATP-grasp) and the histidine acid Phosphatase (Phosphatase) domains within the VIP proteins from *Homo sapiens* (Hs, Genbank AAH57395), *Saccharomyces cerevisiae* (Sc, NP_013514) and *Arabidopsis thaliana* (*AtVip1* Gene ID, 821297; *AtVip2* Gene ID, 831359). The red asterisks denotes in both panels the conserved aspartic acid residue (D) required for kinase activity, and the black dot, the known phosphorylated serine residues within the Arabidopsis VIPs. The lower panel contains the amino acid alignment of the boundary region between the ATP-Grasp and Phosphatase domains. The beginning of the Histidine Acid phosphatase domain is boxed, followed by the PH domain and Arg417 is marked by the blue arrow.

The *Vip* genes are conserved across eukaryotes, including plants (Mulugu et al., 2007; Desai et al., 2014). They have a dual domain structure consisting of an N-terminal ATP grasp domain with kinase activity and a C-terminal histidine acid phosphatase domain or “phytase” domain (Mulugu et al., 2007) (see Figure 4). The human PPIP5K1 phosphatase domain is not active with InsP_5_, InsP_6_, PP-InsP_4_, or PP-InsP_5_ as the substrate or even *p*-nitrophenyl phosphate, a generic substrate for acid phosphatases (Gokhale et al., 2011). This is probably due to the fact that PPIP5Ks lack a conserved histidine essential for phosphatase activity. In addition, the phosphatase catalytic region is interrupted by a Pleckstrin Homology (PH) domain (Gokhale et al., 2011). The PH domain is found in signaling proteins and is responsible for binding phospholipids or molecules derived from their head group (Scheffzek and Welti, 2012). The hybrid PH domain in PPIP5K1 preferentially binds PtdIns(3,4,5)P_3_ and can also bind InsP_6_ allowing PPIP5K1 to translocate from the cytoplasm to the plasma membrane when the PtdIns(3,4,5)P_3_ signaling pathway is activated (Gokhale et al., 2011). Critical to ligand binding is arginine 417 in the PH domain of PPIP5K1 (Gokhale et al., 2011).

All plant species searched through BLAST contained multiple expressed Vip homologs (Desai et al., 2014). Arabidopsis contains two conserved *Vip* genes, *AtVip1* (At3g01310) and *AtVip2* (At5g15070) and the encoded proteins have 94% similarity to each other, but only 50% and 59% similarity to yeast ScVIP1 and human HsVIP1 respectively (Desai et al., 2014). As with yeast and human VIPs, Arabidopsis VIP1 and VIP2 contain a RimK/ATP-Grasp domain (kinase domain) and a histidine acid-phosphatase domain (Figure 4). A BLAST analysis identifies potential *Vip* genes in several other plant species indicating that PPx-InsP synthesis is conserved across the plant kingdom. The kinase domain of the Arabidopsis VIPs contains a conserved aspartic acid (D), which as in yeast and humans, is necessary for activity (Choi et al., 2007; Mulugu et al., 2007; Desai et al., 2014). This residue is also conserved in mouse and Drosophila VIPs, however its role in activity has yet to be confirmed (Fridy et al., 2007). The AtVIP1 and AtVIP2 phosphatase domains are also interrupted with a putative PH domain (Gokhale et al., 2011) (Figure 4), however binding to PtdInsPs has not been tested to date. The arginine residue required for PtdInsP-binding of the human PPIP5K is not conserved in the AtVIPs, however, the substituted lysine at this position provides a similar charge as arginine and there are other arginine residues located nearby (Figure 4). Phosphoproteomics has shown that AtVIPs have a serine adjacent to the phosphatase domain that is phosphorylated. This is not conserved in ScVIP and HsVIP (http://phosphat.mpimp-golm.mpg.de) and may represent a unique mechanism for regulation of VIP activity specific to plants (Desai et al., 2014).

Importantly, both AtVIPs encode catalytically active proteins that allow specific yeast mutants to synthesize InsP_7_ (Desai et al., 2014). There appears to be an intriguing difference in the AtVIPs as compared to the human and yeast VIPs. In the human PPIP5K and yeast VIPs the kinase domain alone is more active than the full-length protein (Fridy et al., 2007; Mulugu et al., 2007), while both full-length AtVIPs are more active than their kinase domains alone (Desai et al., 2014). One explanation for this difference is that the phosphatase domain in the yeast VIP and human PPIP5K may auto-inhibit the kinase activity, and this control may be missing in the AtVIPs. Data from yeast supports this idea of auto-inhibition (Fridy et al., 2007). A second explanation is that the unique presence of a phosphorylated serine in the AtVIPs might provide regulatory control.

Although NMR data on the plant PPx-InsPs is not yet available, it seems reasonable to speculate that only the 1PP-PPx-InsPs should be synthesized in plants, since VIP enzymes are known to phosphorylate at this position. Indeed, the architecture of the VIP catalytic site is what determines the position of phosphorylation (Shears et al., 2013), and as discussed above, 5PP-InsPs may not be synthesized. Intriguing however, both InsP_7_ and InsP_8_ have been found in plants (Desai et al., 2014). This argues for the presence of a plant enzyme capable of 5PP-InsP synthetic ability, since the only structurally verified isomers of InsP_8_ in any organism, 1,5(PP)_2_-InsP_4_ and 5PPP-InsP_5_, both require phosphorylation at a C5 position (Laussmann et al., 1997, 1998; Draskovic et al., 2008; Lin et al., 2009). Further, in yeast, KCS1 and VIP are known to act sequentially to phosphorylate each other's InsP_7_ product, resulting in 1,5(PP)_2_-InsP_4_ synthesis (Figure 2). Alternatively, the plant InsP_8_ molecule may be unique in nature and may not require phosphorylation at the 5-position. Thus, either the plant VIPs are different enough that they can phosphorylate a different position, or there are other enzymes in the plant that can phosphorylate InsP_6_ or InsP_7_. It is important to note one final structural implication of PPx-InsPs: InsP_7_ produced by either IP6K or VIP may not be equivalent since the charge from phosphate is distributed differently in each, and the shapes are not superimposable. As a result, different InsP_7_ enantiomers may interact with different proteins and act in different signaling conditions or pathways.

The similarity of AtVIP1 and AtVIP2 proteins (94% similarity), and the ability of each gene product to restore InsP_7_ synthesis in yeast, suggests that these two genes function similarly at the biochemical level. Thus, the expression patterns of each gene may provide information on where and when InsP_7_ is synthesized. Recent studies showed that *AtVip1* expression is high in vegetative tissues, including shoot of seedlings as well as mature leaf and stem. In contrast, *AtVip2* is most abundantly expressed in roots and reproductive tissues (Desai et al., 2014) suggesting differential spatial regulation. Additionally, since both *AtVip1* and *AtVip2* are expressed together in some vegetative tissues, they may be differentially regulated at a subcellular level. Using subcellular prediction tools, we found compelling predictions for AtVIP1 nuclear localization and an AtVIP2 cytosolic location within the plant cell. Discerning whether the two AtVIPs really do function in these compartments will require experimental validation.

## Meaning of PPx-InsPs: how are they likely to function in plants?

If we continue the analogy of InsP as words, our next challenge will be to understand what these words mean and what information they convey. We will describe how InsP_7_ is known to modify or interact with proteins in other model systems, and how these actions lead to changes in known signaling functions in yeast and animals. Although little is known about PPx-InsPs function in plants and mechanisms regulating inositol signaling differ between plants and animals, it is likely that InsP_7_ conveys plant signaling information by virtue of modifying or interacting with proteins. Drawing parallels from the known and hypothesized roles of InsP_7_ in other organisms, we speculate that PPx-InsPs function in several plant signaling pathways, including, but not limited to energy homeostasis, phosphate (P_i_) sensing, and immune responses. In the following sections, we will elaborate on published data from other model systems that indicates a role for PPx-InsPs in these pathways.

## Energy homeostasis

PPx-InsPs are involved in energy homeostasis both at the cellular and organismal level. Maintaining energy homeostasis, or the balance of intake/production, storage and use of energy often in the form of ATP or sugar, is essential for all living organism. In animals, the AMP Kinase (AMPK) is often named as an energy sensor. Under low energy conditions, AMP is bound, activating the AMP kinase and reprograming the cell to maximize energy acquisition and minimize energy use (Hardie, 2011). In opposition is mammalian Target Of Rapamycin (mTOR), which under high energy conditions promotes growth and cell division (Dunlop and Tee, 2009). In plants, these two enzymes also form the base for maintaining energy homeostasis.

At the cellular level, PPx-InsPs regulate ATP levels through what has been referred to as the “glycolic/mitochondrial metabolic ratio” in yeast (Szijgyarto et al., 2011). Yeast mutants lacking KCS1 have up to five fold higher level of ATP than their wild type controls while overexpression of KCS1 results in a decrease of ATP (Szijgyarto et al., 2011). A similar result is seen with *kcs1* mutant mouse embryonic fibroblast (MEF) cells, where levels of ADP and AMP are low. Further studies showed that the yeast *kcs1* and *kcs1/vip1* mutants as well as MEF *ip6k* mutants have reduction or loss of functional mitochondria. This loss of mitochondrial function with high ATP levels can be explained by an increase in glycolysis and a reduction of ATP used in metabolic pathways. Thus, the lack of InsP_7_ synthesis in these mutants leads to changes in ATP synthesis and utilization. InsP_7_ in this system most likely affects ATP levels by altering transcription of genes that control glycolysis. Specifically, in yeast promoters of glycolytic regulatory genes have a CT-box that binds to the General Control Response 1 (GCR1) transcription factor. InsP_7_ may function to regulate GCR1 directly by a non-catalytic transfer of the β-phosphate from InsP_7_ to an already phosphorylated serine residue in GCR1, resulting in a pyrophosphorylated serine. This modification likely causes a conformational change in GCR1, allowing it to bind the CT-box, thereby regulating the expression of glycolytic regulatory genes (Szijgyarto et al., 2011).

This addition of a new pyrophosphate bond on an already phosphorylated serine residue in a target substrate protein is a proposed mechanism unique to InsP_7_. First demonstrated in yeast, InsP_7_was shown to modify proteins important for ribosomal biogenesis and endosomal trafficking (Saiardi et al., 2004). The pyrophosphorylated serine in target proteins is surrounded by acidic residues, possibly enhancing the recruitment of Mg^2+^ as a cofactor. This modification would be more permanent than phosphorylation by ATP (Bhandari et al., 2007), as no known enzymes exist to remove the pyrophosphate. One limitation to acceptance of this mechanism is that it has been difficult to verify whether such pyrophosphorylated serines occur in *vivo*.

At the whole organism level, InsP_7_ functions in sugar homeostasis through regulating insulin release and glucose uptake in animals. In mammals, InsP_7_ acts as an inhibitor of the Protein Kinase B (also known as Akt) pathway, reducing glucose uptake, insulin sensitivity and protein translation. In response to growth factors, Akt is normally phosphorylated by a protein kinase named PDK1 (3-phosphoinositide-dependent protein kinase 1), which activates the GSK3β (Glycogen synthase kinase 3) and mTOR signaling pathways. Activation of Akt requires binding of its PH domain to PtdIns(3,4,5)P_3_, associated with the plasma membrane. When bound to PtdIns(3,4,5)P_3_, Akt undergoes a conformational change which exposes its activation domain, allowing Akt to be phosphorylated and activated by PDK1 (Calleja et al., 2007). InsP_7_, produced by IP6K1, acts as an inhibitor of Akt by competing for binding to the PH domain within Akt. This effectively prevents the phosphorylation of T308 of Akt (Chakraborty et al., 2010), and dampens Akt signaling. *ip6k1* loss-of-function mutant mice are smaller than their wild type littermates and have lower circulating levels of insulin, but are not diabetic and they have normal blood glucose levels (Bhandari et al., 2008). These genetic data underscore the role of InsP_7_ in global metabolic control.

While insulin is not made by plants, gene homologs functioning in the Akt, GSK3β, and mTOR pathways exist in plants, and have been implicated in growth control pathways. Plants contain homologs of both Akt (i.e., Adi3: AvrPto-dependent Pto-interacting protein 3) and the kinase that activates Akt, PDK1. Most studies indicate that plants do not synthesize PtdIns(3,4,5)P_3_, however plant PDK1 is known to bind phosphatidic acid via its PH domain, allowing membrane localization (Anthony et al., 2004) and the subsequent activation of Adi3 (Devarenne and Martin, 2007). Adi3 can suppress the activity of a major regulator of plant metabolism and AMPK homolog, SnRK1 (Sucrose non-fermenting Related Kinase 1), through phosphorylation of a SnRK1 multiple subunit complex (Avila et al., 2012). GSK-3 kinases are negative regulators of signal transduction pathways controlling metabolism and developmental events across the animal kingdom. In plants, GSK3 homologs are involved in brassinosteroid (BR) signaling pathways. Specifically, the brassinosteroid insensitive 2 (BIN2) protein is a GSK-3 that functions as a negative regulator of BR signal transduction (Yan et al., 2009; Clouse, 2011). As with the animal GSK-3 signaling, BR signal transduction is required for proper metabolic and developmental control throughout the life of a plant. In the case of mTOR, the Arabidopsis gene homologs are known to be important regulators of metabolic changes in response to glucose. Arabidopsis TOR signaling has been linked to transcriptional reprogramming of central and secondary metabolism and other processes within plants (Xiong and Sheen, 2014).

We do not yet know whether InsP_7_ in plants regulates transcription via pyrophosphorylation or whether InsP_7_ can compete with binding to PH domains of plant proteins, however, both are potential mechanisms by which InsP_7_ could act. Determining whether plants use InsP_7_ to regulate metabolism or growth, and the mechanistic details of such regulation will benefit from the development of genetic resources to examine *Atvip* loss-of-function mutants. In addition, we need to know whether PPx-InsPs levels are altered by changes in energy or metabolic status. Answering these questions is now possible as the basis for detecting and measuring PPx-InsPs in plants has been established, and the *AtVip* genes have been cloned and shown to encode active proteins.

## P_i_ sensing

PPx-InsPs are also involved in perceiving and maintaining P_i_ levels and numerous studies link PPx-InsPs to low P_i_ responses in other organisms. Plant P_i_ homeostasis is a highly regulated process (Zhang et al., 2014) and it is important to consider whether PPx-InsPs play a role in this process in plants. P_i_ sensing involves the perception of P_i_ present in the environment, followed by acquisition, remobilization and recycling of P_i_ to maintain P_i_ homeostasis. In yeast, the response to P_i_ starvation is regulated by the P_i_-responsive (PHO) signaling pathway, including the Pho80-Pho85 cyclin-CDK (cyclin dependent kinase) complex (Lenburg and O'shea, 1996; Carroll and O'shea, 2002). When P_i_ levels are low, the Pho80-Pho85 protein complex is inactive. As a result, the Pho4 transcription factor is not phosphorylated and remains in the nucleus where it acts to activate PHO genes (Kaffman et al., 1994; O'neill et al., 1996).

Though there is some lack of consensus for the exact mechanism by which PPx-InsPs control P_i_ sensing, it is clear that they play a role in P_i_ homeostasis. One group found that low P_i_ elevates InsP_7_, and genetic evidence suggested that it was 1PP-InsP_5_, although this was not experimentally confirmed (Lee et al., 2007). This group showed that InsP_7_ physically interacts with Pho81, inactivating the Pho80-Pho85 complex, and ultimately leading to changes in gene expression required to maintain metabolic homeostasis under low P_i_ conditions. In addition, this pathway was dependent on the activity of the yeast VIP genes (Lee et al., 2007). The finding that InsP_7_ is elevated in yeast in response to low P_i_ has been contested by other investigators. Exposure of wild type yeast to P_i_-free medium for 20 min resulted in a decrease of intracellular PPx-InsPs levels by 80%, without affecting InsP_6_ levels (Lonetti et al., 2011).

The change in InsP_7_ levels is not the only phenotype that suggests KCS1 is involved in the yeast low P_i_ response. An intriguing connection between KCS1 and P_i_ sensing comes from recent studies that found that Pho4 binds to intragenic regions of the *Kcs1* gene, promoting the transcription of intragenic and antisense RNA. The authors suggested that the truncated KCS1 protein produced could down-regulate KCS1 function (Nishizawa et al., 2008). An alternative hypothesis is that this truncated KCS1 protein has an altered enzymatic property yet to be determined (Saiardi, 2012). Further work is needed to examine these possibilities and determine the function of intragenic and antisense *Kcs1* RNA. In addition, recent work examining the lipidome of numerous yeast mutants found similarities in changes in sphingolipids of *pho85* and *kcs1*, but not *vip1* mutants, suggesting that similar metabolic changes take place in *pho85* and *kcs1* mutants (Da Silveira Dos Santos et al., 2014). Together these data suggest that either *Vip* or *Kcs1* genes, or possibly both, are linked to P_i_ sensing and sphingolipid homeostasis in yeast.

In animal cells, IP6K has been identified as a stimulator of P_i_ uptake in response to low nutrients. A study found that the mRNAs expressed in rabbit duodenum from a rabbit fed a low P_i_ diet can stimulate Na^+^-dependent P_i_ uptake when injected into *Xenopus* oocytes (Yagci et al., 1992). From this pool of mRNAs, the P_i_ uptake stimulator (PiUS) gene was isolated and confirmed to increase P_i_ uptake when expressed in *Xenopus* oocytes (Norbis et al., 1997). The *PiUS* gene was later found to encode an IP6K, and the gene is now known as *Ip6k2* (Saiardi et al., 1999).

In plants, InsPs are essential for P_i_ response and homeostasis. Arabidopsis mutants in *ipk1*, which catalyzes the addition of a phosphate at the 2-position to select substrates, have a 83% reduction in InsP_6_ levels compared to wild type, and are hypersensitive to P_i_ (Stevenson-Paulik et al., 2005). These mutants have increased uptake of P_i_ and root to shoot translocation of P_i_ (Kuo et al., 2014). Many plant responses to P_i_ starvation (PSR) are regulated at a transcriptional and post-transcriptional level. A recent study has shown that a sub set of PSR genes involved in P_i_ uptake, translocation and remobilization are up regulated in the *ipk1* mutant under P_i_ sufficient conditions (Kuo et al., 2014). Additionally, increased expression of a subset of PSR genes was shown to correlate with a reduction of histone H2A.Z occupancy (Smith et al., 2010) and interestingly, H2A.Z occupancy at chromatin sites associated with several PSR genes was found to be significantly reduced in *ipk1* (under both sufficient and low P_i_) compared to wild type (Kuo et al., 2014). However, Arabidopsis mutants with lower InsP_6_ levels including *mips1* (myo-inositol 1-phosphate synthase), do not show an accumulation of P_i_, indicating that InsP_6_ *per se* is probably not the molecule utilized for sensing P_i_ (Kuo et al., 2014). This implicates other InsPs or the PPx-InsPs as controllers of P_i_ sensing. In particular, since PPx-InsPs are synthesized from InsP_6_ substrates, these molecules might serve as critical players in sensing P_i_. We note that the conversion between InsP_6_ and the PPx-InsPs might be important as InsP_6_ serves an important function in phosphorous storage (Raboy, 2003).

Studies on yeast and animal mutant responses to low P_i_ were among the first to highlight a specific property of InsPs that may be especially important for understanding PPx-InsP function. Response to environmental stress, including P_i_ starvation, requires the fine tuning of TOR and the TORC1 complex activity. This results in the down regulation of ribosomal and protein synthesis regulatory genes, as well as the up regulation of stress response genes (Loewith et al., 2002). Working in parallel with the inactivation of TORC1, the histone deacetylase (HDAC) enzyme is recruited to turn off expression of ribosomal and protein synthesis regulatory genes (Alejandro-Osorio et al., 2009). This HDAC activity is dependent on InsP_4_, which is known to act as a “molecular glue” allowing the HDAC Rpd3L complex to interact with its co-repressor, SMRT (silencing mediator of retinoic acid and thyroid hormone receptor) (Watson et al., 2012). InsP_7_ has been hypothesized to interact with this same complex (Worley et al., 2013), suggesting that PPx-InsPs may play a role in chromatin remodeling thereby regulating gene expression.

There are other known cases of InsPs serving a role as a type of molecular glue, and these bear mentioning. InsP_6_ and InsP_5_ have been found in the auxin (TIR1; Transport Inhibitor Response 1) (Tan et al., 2007) and jasmonic acid receptor, COI1 (Coronatine Insensitive 1) (Sheard et al., 2010; Mosblech et al., 2011), respectively. In both of these examples, InsPs are lodged between the F-Box and the repressor protein target in the E3 ubiquitin ligase complex. When the hormone is present, the repressors for auxin and jasmonic acid, Aux/IAA (Auxin inducible) and JAZ (Jasmonate Zim-domain protein) respectively, are degraded, allowing for transcription of hormone responsive genes. TIR1 is a member of a family of F-box proteins whose five members differ slightly in expression, biochemical activity or function (Parry et al., 2009) and Aux/IAA belongs to an even larger family of 29 proteins (Remington et al., 2004). Like TIR1, COI1 is a member of the F-Box family while JAZ is a 12 member subgroup of TIFY (named for the shared TIF[F/Y]XG motif) (Chini et al., 2007; Vanholme et al., 2007). With all the potential combinations of hormone receptors and repressor proteins, it is interesting to speculate whether other InsPs, including PPx-InsPs, may function as cofactors in hormone signaling to regulate transcription.

## Immune response

The innate immune system is the first line of defense in both plants and animals. The first step in the innate immune response pathway involves the recognition of pathogen-associated molecular patterns (PAMPs) by the host pattern recognition receptors (PRRs) on the cell surface or cytoplasm. In plants, pathogen detection, signaling, and immune response takes place in most cells, while animal immune systems have evolved specialized mobile immune cells. Detection of PAMPs by PRRs initiate signaling cascades which can result in Ca^2+^ release, activation of kinases, and transcription factors, production of reactive oxygen species and alterations in other signaling pathways within the organism (Jones and Dangl, 2006). In animals, RIG-1 (retinoic acid-inducible gene 1) is a PRR in the cytoplasm, which detects double stranded viral DNA and activates a signaling cascade in which the transcription factor IRF3 (Interferon regulatory factor 3) is phosphorylated. IFR3 then moves into the nucleus and promotes the expression of type-1 interferon genes. The interferon proteins then stimulate anti-viral or anti-bacterial activity in leukocytes (Trinchieri, 2010). Recently, this innate response pathway was linked to PPx-InsPs. An *in vitro* study found both InsP_7_ and InsP_8_ are capable of inducing an interferon response through the RIG-1 signaling pathway. The authors of this study speculated that 1PP-InsP_5_ is the physiologically relevant molecule and acts a co-factor for protein interactions or by β-phosphorylation of a serine residue on IRF3, a type of PPx-InsP-driven protein pyrophosphorylation event that we have previously discussed (Pulloor et al., 2014).

The innate immune system in plants and animals share many similarities in the use of PAMPs and PRR as a method of detecting pathogens. Plants have a large diversity of PRR and Resistance (R) genes, however homologs of the RIG-1/IRF3 pathway have not been found in plants. Therefore, while it is interesting to speculate that PPx-InsPs may regulate specific defense transcription factors in plants, the lack of RIG-1 and IRF-3 homologs suggests that this pathway may be unique to animal innate immunity signaling.

A second role of PPx-InsPs in the animal innate immune response involves the afore-mentioned PDK1/Akt signaling pathway. This complex pathway regulates multiple central biological processes including cell survival, proliferation, growth, and metabolism (Hemmings and Restuccia, 2012). In the immune system, neutrophil activation is tightly controlled, with 5PP-InsP_5_ acting as a negative regulator. As described previously, 5PP-InsP_5_ competes for binding with Akt through the PH domain. Upon infection, 5PP-InsP_5_ levels drop allowing Akt to translocate to the membrane and allow for the induction of PtdIns(3,4,5)P_3_ signaling, leading to neutrophil activation and superoxide production. *ip6k* mutant neutrophils have increased bactericidal activity and ROS production (Prasad et al., 2011). Akt triggers reactive oxygen species and nitric oxide production, and is not limited to neutrophils, it can also regulate programmed cell death in other cell types (Lam, 2004). The inhibition of Akt signaling by InsP_7_ is a general phenomenon, however, the mechanism of regulation and the biological outcome may differ depending on tissue and signaling context (Prasad et al., 2011).

A common characteristic of the innate immune response is the programed cell death of infected cells to reduce the spread of disease. In plants, localized programed cell death stimulated by the hypersensitive response occurs rapidly in response to pathogen infection (Morel and Dangl, 1997). As mentioned previously, plants have a homologous pathway to the PDK1/Akt pathway in mammals. In plants, the homolog to Akt is Adi3, which acts in the immune response as a negative regulator of cell death through the MAPK kinase cascade. Akt and Adi3 kinases may be a target for pathogen manipulation of the host In the case of *Pseudomonas* infection of tomato, the bacterial effector protein AvrPto interacts with the Adi3 presumably to manipulate cell death (Devarenne et al., 2006). It is intriguing to speculate whether PPx-InsPs may serve as innate immunity signaling molecules in plants, perhaps by acting to antagonize Adi3 signaling. However it should be noted that although functionally similar, Akt and Adi3 share only 21.4% amino acid identity, suggesting differences in regulation and possibly function (Devarenne et al., 2006).

A final connection between PPx-InsPs and plant innate immune signaling involves plant mutants defective in the synthesis or metabolism of InsPs. Transgenic plants constitutively expressing the human type 1 inositol polyphosphate 5-phosphatase (InsP 5-ptase, the enzyme which dephosphorylates InsP_3_), showed a compromised defense response, including decreased expression of defense genes and a reduction in the systemic acquired immunity in response to a bacterial pathogen (Hung et al., 2014). Furthermore, plants defective in synthesis of *myo*-inositol and InsP_6_ were also more susceptible to disease, including viral, bacterial and fungal pathogen infection (Murphy et al., 2008). It was concluded that InsP_6_ and not its precursors is the critical InsP for this phenotype, however, the authors of this study could not rule out a role for PPx-InsPs in this process due to the difficulty in detection (Murphy et al., 2008). Crops with altered InsP profiles, specifically low InsP_6_, have been developed to combat issues of nutrition and P_i_ pollution (Raboy, 2007). If InsP_6_ or PPx-InsPs play a role in pathogen resistance and immune response, it could negatively impact the performance of these so-called low phytate crops.

## Concluding remarks

PPx-InsPs have recently been identified in higher plants, adding new molecular players in the plant inositol signaling pathway. Both InsP_7_ and InsP_8_ have been detected in a handful of plant species. With two *Vip*/*PPIP5K* gene homologs as the only identified kinase to synthesize PPx-InsPs, the predicted species are 1PP-InsP_5_ and either 1,3(PP)_2_-InsP_4_ or 1PPP-InsP_5_. Further work is needed to identify the stereochemistry of plant PPx-InsPs and to clarify the regulatory components involved in their synthesis and metabolism. Drawing parallels to known roles of PPx-InsPs in other eukaryotes, plant PPx-InsPs may have a role in energy, P_i_ sensing, and innate immunity signaling pathways. Thus, identification of PPx-InsPs in plants presents a new avenue and tool that may be useful for improving crop yield, reduced fertilizer demand, and improved growth under stress.

### Conflict of interest statement

The authors declare that the research was conducted in the absence of any commercial or financial relationships that could be construed as a potential conflict of interest.
